# Vision Improvement with Refractive Correction Does Not Completely Exclude Major Eye Diseases: Analyses of Visually Impaired South Korean Population in the Korea National Health and Nutrition Examination Survey 2009–2011

**DOI:** 10.1155/2017/3412904

**Published:** 2017-11-29

**Authors:** Young-Woo Suh, Ji Sung Lee, Hwan Heo, Shin Hae Park, Seung-Hyun Kim, Key Hwan Lim, Nam Ju Moon, Sung Jin Lee, Song-Hee Park, Seung-Hee Baek

**Affiliations:** ^1^Department of Ophthalmology, Korea University College of Medicine, Seoul, Republic of Korea; ^2^Clinical Research Center, Asan Medical Center, Seoul, Republic of Korea; ^3^Department of Ophthalmology, Chonnam National University Medical School and Hospital, Gwangju, Republic of Korea; ^4^Department of Ophthalmology & Visual Science, College of Medicine, The Catholic University of Korea, Seoul St. Mary's Hospital, Seoul, Republic of Korea; ^5^Department of Ophthalmology, School of Medicine, Ewha Womans University Mokdong Hospital, Seoul, Republic of Korea; ^6^Department of Ophthalmology, College of Medicine, Chung-Ang University Hospital, Seoul, Republic of Korea; ^7^Department of Ophthalmology, Soonchunhyang University Seoul Hospital, Seoul, Republic of Korea; ^8^Department of Ophthalmology, Kim's Eye Hospital, Konyang University College of Medicine, Seoul, Republic of Korea

## Abstract

**Purpose:**

To investigate the association between vision improvement with refractive correction in the visually impaired eyes and the prevalence of ocular comorbidities in the South Korean population.

**Materials and Methods:**

The data of 24,620 individuals in the Korea National Health and Nutrition Examination Survey (KNHANES 2009–2011) were reviewed. Visual impairment was defined as a presenting visual acuity < 20/60. The participants with visual impairment in at least one eye were divided into 3 groups according to the best-corrected visual acuity (group 1: <20/30, group 2: ≥20/30 but <20/25, and group 3: ≥20/25). The prevalence of ocular comorbidities was estimated and compared between the three groups.

**Results:**

Visual impairment in at least one eye was found in 3031 individuals. Groups 1, 2, and 3 comprised 23.5%, 22.2%, and 54.3% of these visually impaired eyes, respectively. The prevalence of cataract, diabetic retinopathy, age-related macular degeneration, corneal opacity, blepharoptosis, and pterygium was similar to or even higher in group 2 compared to group 1. The prevalence of glaucoma and age-related macular degeneration was 5.40% and 11.39%, respectively, in group 2 and 3.31% and 3.76%, respectively, in group 3.

**Conclusions:**

Appropriate ophthalmologic examination is necessary even if people exhibit vision improvement after optical correction.

## 1. Introduction

Visual impairment, which decreases quality of life, increases mortality, and adversely affects socioeconomic state, is a major global health problem [1, 2]. Visual impairment can be defined in several ways. The definition of visual impairment in the international statistical classification of diseases, injuries, and causes of death, 10th revision (ICD-10), H53, was based on best-corrected visual acuity (BCVA) [3]. However, there has been an increasing consensus that the definition based on BCVA is inappropriate because it could underestimate visual impairment caused by an uncorrected refractive error [4, 5]. L. Dandona and R. Dandona noted that 38% of visually impaired people would be erroneously excluded by using the BCVA definition of visual impairment [4]. Considering this perspective, the International Classification of Diseases Update and Revision, 2006, defined visual impairment by using the presenting visual acuity (PVA). PVA is the visual acuity (VA) measured with the currently available refractive correction, if any [5]. According to a WHO report, the most prevalent cause of visual impairment in 2010, as defined by using the PVA, was an uncorrected refractive error (42%) [6]. Visual impairment caused by an uncorrected refractive error could result in loss of educational and employment opportunities, decreased quality of life, and loss of economic gain for individuals and societies [5].

Wearing proper glasses can improve VA in visually impaired patients according to their refractive error. If their VA improves with glasses, their need for further ophthalmologic evaluation would decrease, and they would think they do not have any ocular diseases other than a refractive error. However, they can have ocular comorbidities such as glaucoma and age-related macular degeneration (ARMD), which can preserve normal central vision with refractive correction in the early stage and cause visual loss in the advanced stage. In this aspect, people with normal BCVA can have serious ocular comorbidities. These ocular diseases may need early detection; proper management will prevent visual loss. However, an epidemiologic study on ocular comorbidities of visually impaired patients who improved their vision with glasses has not been conducted. Additionally, the prevalence of ocular comorbidities according to the degree of vision improvement with refractive correction in people with visual impairment has not been fully investigated.

The purpose of this study was to investigate the prevalence of ocular comorbidities in relation to the degree of vision improvement in the visually impaired Korean population (PVA < 20/60) by analyzing the Korea National Health and Nutrition Examination Survey (KNHANES) data.

## 2. Materials and Methods

### 2.1. Study Population

The KNHANES is a cross-sectional nationwide survey that has been conducted by the Korean Centers for Disease Control and Prevention since 1998 to estimate the general health and nutritional status of the South Korean population. The KNHANES uses a stratified, multistage, clustered sampling method for the annual survey results to represent the entire noninstitutionalized South Korean civilian population. Ophthalmologic examinations and interviews have been included in the survey since July 2008.

The data of 25,692 civilian and noninstitutionalized South Korean individuals aged ≥ 5 years who participated in KNHANES IV-3 (2009) and KNHANES V-1, 2 (2010, 2011) were reviewed. The 1072 participants whose ophthalmologic examination data were missing (due to poor cooperation or refusal of eye examination) were excluded. The data of 24,620 subjects with ophthalmologic examination results for at least one eye were included in this study. The study adhered to the tenets of the Declaration of Helsinki and was reviewed and approved by the Kim's Eye Hospital Institutional Review Board.

### 2.2. Ophthalmologic Examinations

The methods utilized for performing ocular examinations and determining ophthalmologic abnormalities in KNHANES were described in detail elsewhere [7]. In brief, distant VA was measured at a distance of 4 m by using an international standard vision chart (Jin's vision chart, Seoul, Korea) [8]. Uncorrected VA, PVA, and BCVA were measured. The PVA was considered the uncorrected VA for subjects who did not wear glasses and the spectacle-corrected VA for those who wore glasses. The refractive errors of all subjects were measured with an autorefractor keratometer (KR 8800; Topcon, Tokyo, Japan). The VA was measured with correction of the refractive error obtained using the autorefractor if the subjects had a PVA of less than 20/25 (8327 subjects). If the corrected VA was less than 20/25, a pinhole was added to measure the pinhole-corrected VA. The best value among the PVA, corrected VA with an autorefractor value, and pinhole-corrected VA was considered as the BCVA.

### 2.3. Definition

Visual impairment was defined based on standard WHO criteria [6]. Low vision was defined as PVA < 20/60 but ≥20/400. Blindness was defined as PVA < 20/400. Visual impairment comprised low vision and blindness (PVA < 20/60). The diagnostic criteria of ocular disorders used in this study are described elsewhere and summarized as follows [7, 9]. Subjects with a spherical equivalent (spherical diopters (D) plus half of the cylindrical D) ≤−0.75 D and ≥+1.00 D were regarded as having myopia and hyperopia, respectively. Subjects with a cylinder ≥ 0.75 D were noted as having astigmatism. Strabismus was measured using the cover-uncover test, prism and alternate cover test, and/or Krimsky test. It was defined as a manifested or latent ocular deviation at a distance or near fixation with or without spectacle correction, which included an esodeviation of ≥10 prism diopters (PD), an exodeviation of ≥15 PD, or any vertical deviations. Subjects were diagnosed with blepharoptosis when their marginal reflex distance (MRD1; the distance between the upper eyelid margin and the corneal light reflex) was 2 mm or less. A slit-lamp examination (Haag-Streit model BQ-900; Haag-Streit AG, Koeniz, Switzerland) was performed. Cataract was categorized into nuclear, cortical, or posterior subcapsular cataract. The presence of pterygium and corneal opacity that precluded obtainment of a nonmydriatic fundus photograph was also observed. Glaucoma was determined as primary open-angle glaucoma, normal-tension glaucoma, or primary angle-closure glaucoma based on the intraocular pressure, peripheral anterior chamber depth, loss of neuroretinal rim, retinal nerve fiber layer defect, and an abnormal visual field as measured using frequency doubling perimetry (Humphrey Matrix, Carl Zeiss Meditec Inc., Dublin, CA, USA). The presence of ARMD was determined using fundus photographs taken by a digital nonmydriatic fundus camera (TRC-NW6S, Topcon) and graded using the grading protocol of the International Age-related Maculopathy Epidemiological Study Group [10]. In subjects who had a history of diabetes mellitus, a random blood glucose level ≥ 200 mg/dl, and/or were suspected of having diabetic retinopathy on the basis of nonmydriatic fundus photographs, seven standard photographs were obtained after pupil dilation. Diabetic retinopathy was defined as the presence of one or more retinal microaneurysms or retinal blot hemorrhages in those subjects. Examinations for cataract, pterygium, corneal opacity, glaucoma, ARMD, and diabetic retinopathy were performed only in patients aged ≥ 19 years.

### 2.4. Data Analysis

The prevalence of ocular comorbidities in the participants with visual impairment in at least one eye was investigated. The PVA of the eye with worse vision was selected for analysis of visual improvement after best refractive correction and ocular comorbidities in the eye with PVA < 20/60 to include monocular visual impairment. When the PVAs were the same in both eyes, the right eye was chosen for the analysis. The eyes with visual impairment (PVA of at least one eye is <20/60) were divided into three groups according to their BCVA, which is the result of refractive correction. The subjects with BCVA < 20/60 (visual impairment), BCVA ≥ 20/60 but <20/25 (subnormal VA), and BCVA ≥ 20/25 (normal VA) were included in groups 1, 2, and 3, respectively. The differences in age distribution were analyzed among the groups by using the chi-squared test. The proportion of each group among subjects with visual impairment was investigated according to the vision correction status at presentation. The prevalence for each ocular comorbidity was expressed as a percentage of the study population with standard error and compared between the groups. Because the KNHANES applied weighted values to allow the survey results to represent the entire Korean population, all analyses were conducted by reflecting the weighted values in the KNHANES by using SAS software version 9.3 (SAS Institute Inc., Cary, NC, USA). *P* values < 0.05 were considered statistically significant.

## 3. Results

Among the 24,620 participants aged ≥ 5 years, representing 45,806,029 individuals in the Korean population, 1308 showed visual impairment in the better-seeing eye (representing 2,264,729 individuals in the Korean population); visual impairment in at least one eye was found in 3031 individuals. The prevalence of ocular comorbidities in these visually impaired eyes (by worse eye PVA) is shown in Table 1. The most prevalent conditions were refractive errors (myopia 71.27% and astigmatism 60.31%) and cataract (40.71%). After correcting the refractive error with an autorefractor value ± pinhole, 54.3% of the participants had a BCVA ≥ 20/25 (group 3) and 22.2% had a BCVA ≥ 20/60 but <20/25 (group 2). The BCVA remained <20/60 in 23.5% of the participants (group 1). The weighted percentages are shown in Table 2.  Table 3 shows age distribution according to groups. The most prevalent age group was >70 years in groups 1 and 2. However, it was 10–18 years in group 3. The mean ages in groups 1, 2, and 3 were 63.7, 63.3, and 34.0 years, respectively, which were significantly different from each other (*P* < 0.0001 by *t*-test).

Among the participants with visually impaired eye(s), 378 wore glasses at presentation. Of these participants, 49.95%, 25.62%, and 24.42% were in groups 1, 2, and 3, respectively (Table 4). About half of subjects who were wearing glasses showed improvement of their vision to subnormal or normal VA after proper optical correction.

The prevalence of ocular morbidities according to groups is shown in Table 5. The prevalence of myopia was significantly higher in group 3 than in groups 1 and 2. However, hyperopia and astigmatism were significantly more prevalent in groups 1 and 2 than in group 3. There was no difference between groups 1 and 2 in the distribution of refractive errors. The prevalences of strabismus and glaucoma were significantly higher in group 1 than in groups 2 and 3. Blepharoptosis was significantly more prevalent in groups 1 and 2 compared to group 3. Corneal opacities were more prevalent in groups 1 and 2 than in group 3, with a statistically significant difference between groups 1 and 3 only. The prevalence of cataract was highest in group 2 and lowest in group 3, and the difference was significant. Pterygium, diabetic retinopathy, and ARMD were more prevalent in groups 1 and 2 than in group 3; the prevalence did not differ significantly between groups 1 and 2. The prevalence of glaucoma and ARMD, which needs early detection to prevent vision loss, was 5.40% and 11.39%, respectively, in group 2 and 3.31% and 3.76%, respectively, in group 3.

## 4. Discussion

In the current study, we investigated ocular comorbidities in the visually impaired eyes according to the improvement of VA after correction of a refractive error. The prevalence of myopia in South Korea was reportedly very high, up to 53.7% [7, 11], with rates of 78.8% in the 12–18-year age group and 75.3% in the 19–29-year age group [7]. In this study, the most common ocular comorbidity in the eyes with PVA-defined visual impairment was myopia (71.27%). Myopia was the only comorbidity that was more prevalent in group 3 than in the other groups. It was also the most prevalent ocular comorbidity in group 3. This indicates the importance of proper myopia correction for visually impaired South Korean individuals to reduce visual impairment. Hyperopia and astigmatism were more prevalent in groups 1 and 2 than in group 3. Because hyperopia and astigmatism are known to induce amblyopia more than myopia, uncorrectable or partially correctable amblyopia could induce visual impairment. However, the prevalences of hyperopia and astigmatism in the Korean population were reportedly 43.8% and 79.4%, respectively, in the 60–69-year age group, which were higher than those in younger age groups [7]. In this study, the mean ages of groups 1 and 2 were 63.7 and 63.3 years, respectively, compared to 34.0 years of group 3. Therefore, the higher prevalence can be explained partly by the older ages of groups 1 and 2 and partly by amblyopia. The prevalences of strabismus and glaucoma were significantly higher in group 1 than in groups 2 and 3. Although the prevalences of strabismus in groups 2 and 3 were similar to those in the general Korean population (1.5%) [7], group 1 had a significantly higher prevalence (10.01%). Both strabismus-induced amblyopia and sensory strabismus due to visual impairment should be considered. Strabismus can be either a cause or a result of visual impairment, and this could not be determined from these cross-sectional data. Glaucoma is also more prevalent in the older age group, but the prevalence in group 1 (11.93%) was much higher than the reported prevalence in the 60–69-year age group from the KNHANES population (2.2%) [7]. Glaucomatous optic nerve damage can be one of the most prevalent ocular comorbidities that induce BCVA-defined visual impairment.

In groups 2 and 3 where the VA of the participants can be improved to ≥20/60 with proper optical correction, these participants also had numerous ocular comorbidities. In group 2, cataract was the most prevalent ocular comorbidity excluding refractive errors. The prevalences of blepharoptosis (24.97%), cataract (70.69%), pterygium (11.12%), glaucoma (5.5%), and ARMD (11.39%) in group 2 were higher than the reported prevalence for the general Korean population (11.0%, 2.1%, 5.4%, 1.4%, and 5.1%, resp.) [7]. Considering the mean age of group 2 (63.3 years), the prevalences of glaucoma and ARMD are still higher in this group than in the reported Korean prevalence in the 60–69-year age group (2.2% and 8.8%, resp.). In group 3, cataract was also the most prevalent ocular comorbidity excluding refractive error. Considering the mean age of 34.0 years in group 3, the prevalences of cataract (19.89%), pterygium (2.46%), and glaucoma (3.31%) were higher than the reported Korean prevalence in the 30–39 age group (2.5%, 0.9%, and 0.7%, resp.). These findings imply that even though VA improves to ≥20/60 and >20/25 after best correction in patients with PVA-defined visual impairment, numerous ocular comorbidities could be present other than refractive errors. Among these ocular comorbidities, glaucoma and ARMD can induce uncorrectable visual impairment. Early detection and treatment can prevent or minimize vision loss. Glaucoma does not produce visual symptoms until extensive visual field loss becomes evident in the advanced stage because the central visual field is usually preserved at its early stage. Glaucomatous optic nerve damage is irreversible, and early treatment is known to reduce the risk of glaucoma progression [12]. ARMD is reportedly a leading cause of visual impairment in developed countries [13, 14]. Timely intervention is known to reduce the development of legal blindness or visual impairment caused by neovascular ARMD [15]. Patients with glaucoma or ARMD can improve their vision with glasses if they have a refractive error. The current study showed that even when VA improves to a subnormal or normal level after correction for refractive error, a proper ophthalmologic examination should be performed to detect ocular comorbidities, which need early detection and timely intervention.

For subjects with visual impairment who are already wearing glasses, vision improvement with further refractive correction may be limited. Visual improvement after best correction in these subjects may indicate that their original refractive errors were not properly corrected. About half of the subjects wearing glasses showed vision improvement to ≥20/30, and about half of them showed improvement to ≥20/25. This suggests that additional refractive correction through proper ophthalmologic examination would be necessary in subjects with visual impairment even when they are already wearing glasses.

Because quality of life would be much worse with binocular visual impairment than with monocular visual impairment, most studies defined visual impairment by the PVA or BCVA of the better-seeing eye. In this study, the participants with a PVA < 20/60 in the worse eye were included. Participants with monocular visual impairment were therefore included because we intended to analyze the association between vision improvement by correction and ocular comorbidities in the eyes with PVA < 20/60, not the prevalence of visual impairment per se or ocular comorbidities in the whole South Korean population. Furthermore, the presence of either monocular or binocular visual impairment would not have affected the results of this analysis. When the eyes with visual impairment had a BCVA ≥ 20/60, we divided them into two groups (groups 2 and 3). In the resolution of the International Council of Ophthalmology (ICO) on visual terminology, ICO defined normal vision as VA ≥ 20/25 and mild vision loss as VA < 20/25 but ≥20/30 [16]. Further, ICO reported that people with mild vision loss showed reduced reading distance and had no reserve for small print compared to people with normal vision. Another study also defined VA < 20/25 but ≥20/30 as subnormal VA [17]. We divided the subjects into two groups based on this definition because the distribution of ocular comorbidities that would reduce BCVA can be different between those with normal and subnormal BCVA. After best refractive correction, more than half of the visually impaired eyes showed normal vision (≥20/25). However, 20% were still visually impaired after best correction.

This study used a very large sample from a nationwide survey in South Korea, which represents the entire Korean population, and assessed the ocular comorbidities in visually impaired subjects according to the degree of visual improvement after refractive error correction. However, this study had several limitations. First, the contribution of ocular comorbidities to a decrease in VA could not be conclusively determined from the KNHANES data. Thus, we could not conclude if the major eye disease was the cause of visual impairment. The KNHANES data only provided the VA and coexisting ocular abnormalities without assessing the cause of visual impairment. A further epidemiologic survey that includes an assessment of the major cause of visual impairment in subjects with multiple ocular comorbidities is needed. Second, in KNHANES, the BCVA was obtained by correction with an autorefractor value with or without pinhole addition, and the cycloplegic refraction was not performed. Therefore, this value might be different from that obtained from an ophthalmologic clinic in which manifest/cycloplegic refraction and fine adjustment of refractive correction is used. This could overestimate the prevalence of BCVA-defined visual impairment and underestimate the degree of visual improvement after refractive correction in participants with PVA-defined visual impairment. These factors should be considered when interpreting our study results.

## 5. Conclusions

In conclusion, proper refractive correction would resolve visual impairment of at least one eye in more than half of people with visual impairment in South Korea. Cataract is the most common ocular comorbidity in the visually impaired eyes with VA that remain in the range of visual impairment after refractive correction. Even when a visually impaired eye demonstrates an improvement of VA to a subnormal or normal level, an appropriate ophthalmologic examination is needed to find ocular comorbidities because ocular comorbidities are still prevalent in these individuals.

## Figures and Tables

**Table 1:** Prevalence of ocular comorbidities in the eyes with visual impairment.

Ocular comorbidities	*N*	%	SE
Refractive error			
Myopia	1831	71.27%	(1.14)
Hyperopia	572	15.27%	(0.78)
Astigmatism	1824	60.31%	(1.17)
Strabismus	108	3.34%	(0.42)
Blepharoptosis	515	14.09%	(0.97)
Cataract	1266	40.71%	(1.50)
Pterygium	224	7.05%	(0.60)
Corneal opacities	52	12.90%	(2.17)
Glaucoma	136	5.49%	(0.58)
Diabetic retinopathy	69	2.92%	(0.46)
Age-related macular degeneration	168	6.85%	(0.60)

The presence of cataract, pterygium, corneal opacities, diabetic retinopathy, and age-related macular degeneration was evaluated only in participants aged ≥ 19 years. The data are based on the eyes with worse presenting visual acuity. *N*: number; SE: standard error.

**Table 2:** Improvement of visual acuity after correction in participants with visually impaired eye(s).

Best-corrected visual acuity	*N*	%	Weighted %
<20/60 (group 1)	713	23.5	20.5
≥20/60 and <20/25 (group 2)	672	22.2	18.2
≥20/25 (group 3)	1646	54.3	59.9
Total	3031	100.0	100.0

The data are based on the eye with worse presenting visual acuity. *N*: number.

**Table 3:** Age distribution according to visual improvement after refractive correction.

	Best-corrected visual acuity									
	<20/60 (group 1)			≥20/60 and <20/25 (group 2)			≥20/25 (group 3)			*P* value
	*N*	%	SE	*N*	%	SE	*N*	%	SE	
Age (yrs)										<.0001
5–9	7	0.77%	(0.30)	23	3.04%	(0.74)	106	4.64%	(0.64)	
10–18	5	0.79%	(0.41)	15	3.49%	(1.16)	391	25.30%	(1.33)	
19–29	17	5.53%	(1.58)	8	3.13%	(1.17)	229	20.47%	(1.35)	
30–39	30	5.85%	(1.19)	14	3.00%	(0.91)	195	12.57%	(1.10)	
40–49	43	9.27%	(1.72)	21	6.08%	(1.43)	183	13.00%	(0.99)	
50–59	68	12.87%	(1.66)	46	8.81%	(1.50)	181	10.31%	(0.89)	
60–69	143	15.80%	(1.48)	151	20.02%	(1.81)	185	7.35%	(0.64)	
70–	400	49.12%	(2.69)	394	52.43%	(2.38)	176	6.35%	(0.62)	

*P* value was obtained using the chi-squared test. Visual impairment was based on the eye with worse presenting visual acuity. *N*: number; SE: standard error.

**Table 4:** The proportion of each group according to the vision correction status at presentation.

	Group 1 (*N* = 713)			Group 2 (*N* = 672)			Group 3 (*N* = 1646)		
Presence of vision correction at presentation	Number	Row%	SE	Number	Row%	SE	Number	Row%	SE
No vision correction (*N* = 2627)	513	16.86%	(1.04)	562	17.41%	(0.91)	1552	65.73%	(1.40)
Wearing glasses (*N* = 378)	191	49.95%	(3.13)	101	25.62%	(2.63)	86	24.42%	(2.87)
History of refractive surgery (*N* = 23)	8	23.20%	(9.27)	8	34.66%	(11.94)	7	42.15%	(14.66)
No response (*N* = 3)	1	36.18%	(28.29)	1	33.35%	(27.26)	1	30.48%	(25.96)

Visual impairment was based on the eye with worse presenting visual acuity.

**Table 5:** Prevalence of ocular comorbidities, according to visual improvement after correction.

	Group 1 (*N* = 713)			Group 2 (*N* = 672)			Group 3 (*N* = 1646)			*P* value		
	*N*	%	SE	*N*	%	SE	*N*	%	SE	*P* ^12^	*P* ^13^	*P* ^23^
Myopia	221	44.26%	(3.03)	288	47.89%	(2.56)	1309	85.09%	(1.00)	0.3633	<.0001	<.0001
Hyperopia	138	24.91%	(2.50)	214	28.45%	(2.08)	216	8.91%	(0.75)	0.2896	<.0001	<.0001
Astigmatism	404	74.93%	(2.78)	549	81.18%	(1.84)	854	50.41%	(1.48)	0.0613	<.0001	<.0001
Strabismus	69	10.01%	(1.47)	10	1.40%	(0.49)	23	1.51%	(0.41)	<.0001	<.0001	0.8631
Blepharoptosis	200	26.56%	(2.34)	169	24.97%	(2.13)	128	6.02%	(0.74)	0.5502	<.0001	<.0001
Cataract	425	56.74%	(2.47)	477	70.69%	(2.39)	325	19.89%	(1.44)	<.0001	<.0001	<.0001
Pterygium	88	12.52%	(1.75)	80	11.12%	(1.31)	47	2.46%	(0.41)	0.5133	<.0001	<.0001
Corneal opacities	40	16.08%	(2.89)	5	7.48%	(4.39)	1	2.36%	(2.36)	0.1935	0.0197	0.2746
Glaucoma	60	11.93%	(1.82)	36	5.40%	(1.10)	40	3.31%	(0.66)	0.0014	<.0001	0.0869
Diabetic retinopathy	31	6.38%	(1.40)	24	4.53%	(1.15)	13	1.20%	(0.40)	0.3108	<.0001	0.0003
Age-related macular degeneration	55	11.57%	(1.74)	58	11.39%	(1.77)	54	3.76%	(0.60)	0.6433	<.0001	<.0001

The presence of cataract, pterygium, corneal opacities, diabetic retinopathy, and age-related macular degeneration was evaluated only in participants aged ≥ 19 years. *P*^12^: comparison between groups 1 and 2. *P*^13^: comparison between groups 1 and 3. *P*^23^: comparison between groups 2 and 3. *P* value by chi-squared test.
